# Altered Microstructural Caudate Integrity in Posttraumatic Stress Disorder but Not Traumatic Brain Injury

**DOI:** 10.1371/journal.pone.0170564

**Published:** 2017-01-23

**Authors:** Dana Waltzman, Salil Soman, Nathan C. Hantke, J. Kaci Fairchild, Lisa M. Kinoshita, Max Wintermark, J. Wesson Ashford, Jerome Yesavage, Leanne Williams, Maheen M. Adamson, Ansgar J. Furst

**Affiliations:** 1 War Related Illness and Injury Study Center (WRIISC), Veterans Affairs Palo Alto Health Care System (VAPAHCS), Palo Alto, United States of America; 2 Department of Psychiatry and Behavioral Sciences, Stanford University School of Medicine, Palo Alto, United States of America; 3 Department of Radiology, Harvard University, Cambridge, United States of America; 4 Sierra Pacific Mental Illness Research Education and Clinical Center (MIRECC), Veterans Affairs Palo Alto Health Care System (VAPAHCS), Palo Alto, United States of America; 5 Psychology Service, Veterans Affairs Palo Alto Health Care System (VAPAHCS), Palo Alto, United States of America; 6 Department of Radiology, Stanford University School of Medicine, Palo Alto, United States of America; 7 Department of Neurology and Neurological Sciences, Stanford University School of Medicine, Palo Alto, United States of America; 8 Defense Veterans Brain Injury Center (DVBIC), Veterans Affairs Palo Alto Health Care System (VAPAHCS), Palo Alto, United States of America; Central Institute of Mental Health, GERMANY

## Abstract

**Objective:**

Given the high prevalence and comorbidity of combat-related PTSD and TBI in Veterans, it is often difficult to disentangle the contributions of each disorder. Examining these pathologies separately may help to understand the neurobiological basis of memory impairment in PTSD and TBI independently of each other. Thus, we investigated whether a) PTSD and TBI are characterized by subcortical structural abnormalities by examining diffusion tensor imaging (DTI) metrics and volume and b) if these abnormalities were specific to PTSD versus TBI.

**Method:**

We investigated whether individuals with PTSD or TBI display subcortical structural abnormalities in memory regions by examining DTI metrics and volume of the hippocampus and caudate in three groups of Veterans: Veterans with PTSD, Veterans with TBI, and Veterans with neither PTSD nor TBI (Veteran controls).

**Results:**

While our results demonstrated no macrostructural differences among the groups in these regions, there were significant alterations in microstructural DTI indices in the caudate for the PTSD group but not the TBI group compared to Veteran controls.

**Conclusions:**

The result of increased mean, radial, and axial diffusivity, and decreased fractional anisotropy in the caudate in absence of significant volume atrophy in the PTSD group suggests the presence of subtle abnormalities evident only at a microstructural level. The caudate is thought to play a role in the physiopathology of PTSD, and the habit-like behavioral features of the disorder could be due to striatal-dependent habit learning mechanisms. Thus, DTI appears to be a vital tool to investigate subcortical pathology, greatly enhancing the ability to detect subtle brain changes in complex disorders.

## Introduction

Posttraumatic stress disorder (PTSD) and traumatic brain injury (TBI) are a common occurrence in Veterans. The prevalence of PTSD in Veterans ranges from 10–30% [1, 2] and 15–30% for TBI [3, 4]. In addition, a common sequelae of both PTSD and TBI are impairments in memory. The memory systems in the medial temporal lobe and basal ganglia have different properties. The medial temporal lobe supports declarative memory, which is the conscious recollection of facts and events, while the basal ganglia support nondeclarative memory, which is nonconscious and is implicitly learned through performance rather than recollection [5]. Furthermore, it is not well understood how the different memory systems (declarative vs non-declarative memory) interact in the brain with each other [6, 7], but it is possible that both systems are involved in the pathology of PTSD and TBI, as summarized below.

In terms of regions underlying the medial temporal lobe and basal ganglia memory systems, there has been an extensive body of research addressing functional and volumetric abnormalities in PTSD. Studies have shown that PTSD patients tend to perform poorly on declarative memory tasks compared to controls (for a review see [8]), suggesting a functional impairment of the hippocampal-dependent declarative memory system. There are also studies demonstrating reduced hippocampal volume in patients with PTSD [9–11], though this finding is not consistent [12–14]. Supporting a role of the striatum in PTSD pathology, one study found reduced [15], while another study [16] found increased blood flow to this region in patients with PTSD compared to controls. Similarly, the fMRI literature has been mixed. Several fMRI studies suggest increased activation in the striatum [17, 18], while other studies demonstrated decreased activation [19, 20].

Similarly, patients with traumatic brain injury (TBI) also display memory impairments that could be due to structural and functional abnormalities of the hippocampus and striatum. Neuroimaging and post-mortem neuropathological studies, as well as data from animal models have shown that the hippocampus is highly susceptible to the effects of TBI [21–24], as well as the striatum [23, 25–27] (for a review see [28]). Furthermore, studies have found correlations between memory impairment and learning deficits with hippocampal atrophy, striatal atrophy and decreased activation, and fornix damage in patients with TBI [22, 29–32].

Based upon the evidence presented above, gray matter damage is a relevant feature in PTSD and TBI. Understanding the neuroanatomical basis of functional abnormalities of memory requires understanding whether there are any disruptions in macrostructural and microstructural integrity. While volume is often used to characterize macrostructural integrity, another modality, diffusion tensor imaging (DTI), is a novel tool that may capture microstructural abnormalities in PTSD and TBI. DTI is a non-invasive magnetic resonance imaging technique that provides information about the microstructural architecture in different cerebral tissues in vivo. DTI measures the random molecular motion of water molecules (i.e. diffusion) by applying multiple diffusion-encoding gradients in different orientations.

There are four traditional DTI metrics that are calculated. Fractional anisotropy (FA), which quantifies the directionality of diffusion, is a non-specific biomarker of microstructural architecture and neuropathology and can be regarded as a summary measure for microstructural integrity [33]. Mean diffusivity (MD) measures the average diffusion in all directions and represents isotropic diffusivity, which provides information about changes in the interstitial space and is sensitive to cellular damage (e.g. edema and necrosis) [33]. More neurobiological specificity is available from two directional diffusivities: axial diffusivity (AD), which measures diffusion parallel to the axonal fibers, is correlated with axonal injury [34] or axonal pruning [35], while radial diffusivity (RD), which measures diffusion perpendicular to the fibers, is related to myelin injury or decreased myelination [36].

There is cumulating evidence that DTI is a useful tool to detect subtle microscopic brain tissue alterations before neuronal degradation and atrophy are detectable on a macroscopic level [37, 38]. DTI is traditionally used for characterizing microstructural properties of white matter tracts, but has recently been employed to detect microstructural abnormalities and overall cellular dysfunction in subcortical gray matter regions [14, 39–49]. The validity of DTI for subcortical gray matter regions is due to the high directionality of diffusion in these deep gray matter structures [50]. While there have been various studies examining white matter DTI structural abnormalities in PTSD and TBI [51–53], to our knowledge, there have been no studies examining gray matter microstructural abnormalities in these patient populations in these regions.

PTSD and TBI are frequently referred to as the “signature wound” of the Iraq and Afghanistan wars. Returning Veterans have higher incidences of comorbid PTSD and TBI, as well as other psychiatric illness such as depression [54–56]. However, in terms of gray matter abnormalities in individuals with comorbid PTSD and TBI, the literature is inconsistent. One study [57] found a decrease in cortical thickness, while other studies have not found significant changes in gray matter [58](for a review see [28]). While it is important to look at comorbid conditions, understanding the two separately provides unique insights that can help in targeting treatments with varying degrees of each condition. Examining these pathologies separately may help to understand the neurobiological basis of PTSD and TBI independently of each other. Since both PTSD and TBI are associated with impairments across several domains of memory, we wanted to investigate the neural basis of both of these systems, choosing representative regions in these memory systems due to their role in cognition, (i.e., the hippocampus and caudate). Thus, we investigated whether a) PTSD and TBI are characterized by subcortical structural abnormalities by examining gray matter DTI metrics and gray matter volume of the hippocampus and caudate and b) if these abnormalities were specific to PTSD versus TBI. In order to investigate these aims, we examined three groups of Veterans: Veterans with PTSD, Veterans with TBI, and Veterans with neither PTSD nor TBI (Veteran controls; VC).

## Materials and Methods

### Participants

Eighty-nine Veterans were seen at the War Related Illness and Injury Study Center (WRIISC CA), Veterans Affairs Palo Alto Health Care System (VAPAHCS), a tertiary care clinic that provides clinical evaluations for Veterans with deployment-related health concerns. Veterans referred to the WRIISCs often have one or more of the following presentations: (1) deployment-related health conditions, (2) complex health conditions with no known cause (medically unexplained symptoms), and (3) chronic multi-symptom illness. We examined a sample of 25 Veterans with PTSD (mean age: 47.68; SD: 11.74), 33 Veterans with TBI (mean age: 48.70; SD: 9.48), and 31 Veterans without PTSD or TBI (mean age: 45.03, SD: 10.73) (Table 1). Drug information about the participants was not available. All aspects of the study were approved by the Stanford University and VAPAHCS Institutional Review Board, and written informed consent to analyze clinical data was obtained from all participants.

**Table 1:** Participant demographics.

Demographics	Veterans with PTSD (N = 25)	Veterans with TBI (N = 33)	Veterans without PTSD or TBI (N = 31)
Age (years)	47.68 ± 11.74 (29–70)	48.70 ± 9.48 (28–67)	45.03 ± 10.73 (30–71)
Gender (male/female)	20/5	31/2	29/2
Education (years)	13.08 ± 2.40^*^ (6–18)	14.82 ± 2.27 (11–22)	14.52 ± 2.47 (12–21)
Handedness (left/right/both)	1/20/4^*^^§^	0/33/0	3/28/0
Deployment (yes/no)	25/0	26/7^ǂ^	31/0

Demographics of Veterans with PTSD, with TBI, and without TBI or PTSD (VC). The table displays the mean, standard deviation, range, and count when appropriate.

* p < 0.05, PTSD < TBI.

§ p < 0.05, PTSD < VC.

ǂ p < 0.05, TBI > PTSD & TBI > VC.

### Clinical measures and evaluations

The Clinician Administered PTSD Scale (CAPS) [59] was used to assess PTSD symptoms. PTSD status was assigned a binary score (no or yes). The CAPS is considered the “gold standard” for the assessment of PTSD and has very good psychometric properties across a wide variety of clinical populations and research settings [60]. Only Veterans who met the criteria for PTSD and without a history of TBI were included in the PTSD only group. An exam by a neurologist or a review of patient records was used to determine TBI status. TBI status and severity was determined according to DoD/DVA criteria assessing Alteration of Consciousness (AOC), Loss of Consciousness (LOC), and Post-traumatic amnesia (PTA) [61]. Veterans with mild to moderate TBI were included in the TBI only group (four out of the 33 individuals in the TBI group had a moderate TBI). No individuals with severe TBI were included in our analysis, as these patients are rarely referred to our clinic.

### Cognitive assessment

Each Veteran received a cognitive screening battery as part of a comprehensive medical evaluation. Estimated premorbid intellectual functioning (eFSIQ) was based on a reading recognition measure, the Wechsler Test of Adult Reading (WTAR; [62]). Executive functioning abilities assessed included attention and working memory for auditorily presented numbers (Wechsler Adults Intelligence Scale–Fourth Edition Digit Span subtest; [63]), and processing speed, attention, and set-shifting (Trails A & B; [64]). The Repeatable Battery for the Assessment of Neuropsychological Status (RBANS; [65]) assessed general cognitive functioning. The RBANS is composed of five composite summary indexes: immediate memory, visuospatial/constructional, attention, language, and delayed memory. Examination of Veteran memory performance was based on immediate and delayed memory index values, along with the individual subtests comprising each index: list learning, list recall, figure recall, story learning, and story recall.

### MRI acquisition

Clinical neuroimaging was conducted at the Veterans Affairs Palo Alto Health Care System (VAPAHCS) using a 3T GE Discovery MR750 scanner with an eight channel, GE head coil. High-resolution T1-weighted images were acquired using a three-dimensional spoiled-gradient recalled acquisition (3D-SPGR) in steady state (272 axial slices, repetition time = 7.3 ms; echo time = 3.0 ms; flip angle = 11°; field of view = 250 mm; slice thickness = 1.2 mm with 0.6 between slices; acquisition matrix = 256 × 256; number of excitations = 1.0; voxel dimensions: 1.05 mm × 1.05 mm × 0.60 mm). DTI images were acquired through Array Spatial Sensitivity Encoding (ASSET) Echo Planar Imaging (EPI), slice thickness = 2.0 mm, TR = 6600, TE = 84, in-plane-resolution = 0.94 x 0.94 mm, Flip Angle = 90°, NEX = 1, 30 diffusion-encoding directions at b = 1000 s/mm2, 5 acquisitions at b = 0. This sequence was acquired twice to improve signal-to-noise. The images were read by a neuroradiologist in the weekly WRIISC consensus conference comprised of clinical psychologists, neurologists, nurses, psychiatrists, and a social worker.

### Volumetric processing and analysis

Automated volumetric segmentation was performed using the FMRIB’s Integrated Registration and Segmentation Tool (FIRST) as implemented in FSL [66]. First, each subject’s T1 image was spatially transformed to the MNI 152 standard space using 12 degrees of freedom affine registration as implemented in FLIRT. After subcortical registration, a sub-cortical mask is applied, to locate the different subcortical structures, followed by segmentation based on shape models and voxel intensities. Absolute volumes of subcortical structures are calculated. In order to calculate normalized volumes, FSL’s SIENAX was run [67, 68]. SIENAX starts by extracting brain and skull images from the single whole-head input data [69]. The brain image is then affine-registered to MNI152 space using the skull image to determine the registration scaling [70, 71]. The volumetric scaling factor to be used as normalization for head size is derived from the normalization matrix. All normalized volumes for the hippocampus and caudate are reported.

### DTI processing and analysis

Data processing was conducted using (FMRIB) Software Library (FSL) version 5.0.8 and FMRIB’s Diffusion Toolbox (FDT) [72]. Head motion and eddy current induced distortions were corrected through affine registration of the diffusion-weighted images to the first B0 image. Motion artifacts were inspected and subjects with greater than 3mm of movement were excluded (2 VC subjects). The gradient directions were corrected according to the rotation parameters. Next, non-brain tissue was removed using the Brain Extraction Tool (BET) [69]. The DTIFIT tool was then used to fit a diffusion tensor model to the raw diffusion data at each voxel to get DTI derived metrics (i.e. FA, MD, RD, and AD), fitting the model with weighted least squares. All subjects' DTI data were then aligned into a common MNI space using the nonlinear registration tool FNIRT, which uses a b-spline representation of the registration warp field [73–75]. Next, based on an *a priori* hypothesis, two pairs of ROIs derived from subject-specific masks from FSL’s FIRST tool were nonlinearly aligned into a common MNI space; these ROIs included the left and right caudate and hippocampus. DTI metrics from these subject-specific gray matter ROIs were obtained and used in the analysis. Subject-specific masks rather than standard space atlas masks were used to decrease risk of data contamination of surrounding CSF and white matter.

### Statistical analyses

Data were analyzed using SPSS (V.21, SPSS, Chicago) and R (www.r-project.org). Normality for individual variables was determined by the Shapiro-Wilk test. Depending on normality, variables were analyzed using parametric models or log transformed before running parametric models. Group differences for demographic data and neuropsychological data were analyzed using one-way AVOVA with post-hoc bonferroni correction and chi-square tests when appropriate. Group differences in DTI metrics and volume were analyzed using a univariate GLM model controlling for education, handedness, and deployment with post-hoc bonferroni correction. The first group analysis examined the ROIs bilaterally, while the second group analysis examined the left and right ROIs independently. In order to determine if it was valid to combine the ROIs a multivariate ANOVA (MANOVA) was run. Because MD is a measure that provides information about changes in the interstitial space and cellular damage [33], several recent studies [14, 39–49] have employed MD to investigate abnormalities in subcortical gray matter regions. As such, MD was our primary outcome measure. However, we report all DTI measures and group results for completeness. In order to see if DTI and volume are sensitive to age differences and cognitive measures, correlations between these metrics and age, MD, and volume collapsed across group were carried out. These correlations controlled for education, handedness, and deployment.

## Results

### Demographic group differences

There were no differences in age or gender among Veterans with PTSD, TBI or without PTSD or TBI. There were differences among the groups for education, F(2, 86) = 4.156, p = 0.019. Post-hoc analyses applying a Bonferroni correction revealed the PTSD group (M = 13.08, SD ± 2.40) had less number of years of education than the TBI group (M = 14.82, SD ± 2.27), p = 0.021. There were no differences in years of education between the PTSD and VC groups, or for the TBI and VC groups, *p’s* > 0.05. There was also a significant group difference in deployment and handedness, X^2^ (2) = 12.89, p = 0.002 and X^2^ (4) = 14.217, p = 0.007. For deployment, the TBI group had a greater number of subjects that were not deployed. For handedness, the PTSD group had more subjects that were ambidextrous than the other groups (Table 1).

### Cognitive assessment group differences

There were group differences for eFSIQ, F(2,78) = 3.324, p = 0.045. Specifically, WTAR values suggested higher estimated premorbid intellectual functioning in the VC group as compared to the PTSD group (p = 0.049). There were no group differences for eFSIQ between the TBI group and the VC group or with the PTSD group, *p’s* > 0.05. However, there were differences in declarative memory measures of list learning raw scores, F(2, 59) = 4.324, p = 0.018, list recall raw scores, F(2, 59) = 4.303, p = 0.018, and story recall raw scores, F(2, 59) = 4.938, p = 0.010. For list learning and list recall, post-hoc analyses revealed that the PTSD group performed worse than the VC group, p = 0.033 and p = 0.014 respectively. For story recall, the PTSD group did worse than the TBI group, p = 0.011 and a trend to do worse than the VC group, p = 0.069 (Table 2).

**Table 2:** Participant Cognitive assessment.

Cognitive Assessment	Veterans with PTSD (N = 25)	Veterans with TBI (N = 33)	Veterans without PTSD or TBI (N = 31)
eFSIQ	100.14 ± 10.67^§^	105.63 ± 10.10	106.9 ± 8.49
List Learning (raw)	23.9 ± 5.5^§^	24.3 ± 4.9	27.83 ± 4.2
List Recall (raw)	3.79 ± 2.8^§^	5.10 ± 2.47	6.09 ± 2.33
Story Recall (raw)	7.00 ± 3.02*	9.50 ± 2.01	8.87 ± 2.63

Cognitive assessment of Veterans with PTSD, with TBI, and without TBI or PTSD (VC).

* p < 0.05, PTSD < TBI.

§ p < 0.05, PTSD < VC.

### DTI group differences

#### MD

**Fig 1:**
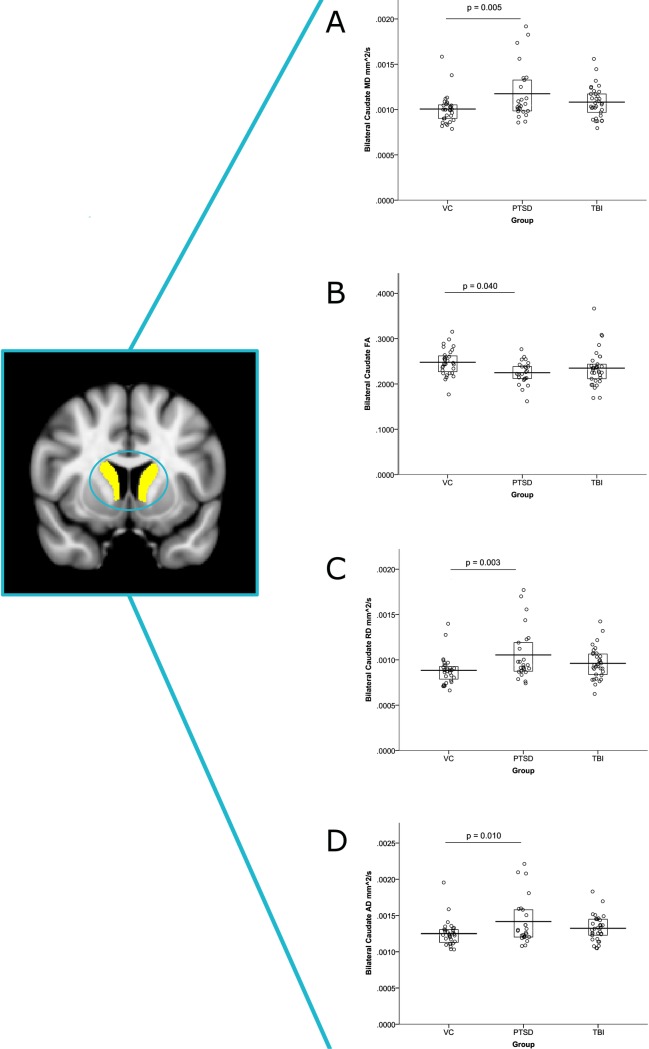
Group differences in MD, FA, RD, and AD. Average MD, FA, RD and AD from the bilateral caudate was compared between the three groups. The bilateral caudate was anatomically defined from the FSL Harvard-Oxford atlas in standard MNI space. Differences between the groups emerged for the PTSD group to have (A) greater MD than the VC group (p = 0.005, (B) lower FA than the VC group (p = 0.040), (C) greater RD than the VC group (p = 0.003), and (D) greater AD than the VC group (p = 0.010) after controlling for education, handedness, and deployment. There were no significant differences for the TBI group, *p’s* > 0.05.

There were group differences in the bilateral caudate, F(5, 83) = 5.350, p = 0.007, for the PTSD group to have higher MD values than VC, p = 0.005 (Fig 1A). In addition, there were differences between the groups in the left caudate, F(5, 83) = 4.610, p = 0.013, and right caudate, F(5, 83) = 5.205, p = 0.007, for the PTSD group to have higher MD values than VC, p = 0.010 and p = 0.006 respectively. There were no significant differences for the TBI group, *p’s* > 0.05.

#### FA

There were group differences in the bilateral caudate, F(5, 83) = 3.204, p = 0.046, for the PTSD group to have lower FA values than VC, p = 0.040 (Fig 1B). There were no other differences among the groups for FA.

#### RD

There were group differences in the bilateral caudate, F(5, 83) = 5.727, p = 0.005, for the PTSD group to have higher RD values than VC, p = 0.003 (Fig 1C), and a trend for the TBI group, p = 0.095. In addition, there were differences between the groups in the left caudate, F(5, 83) = 4.966, p = 0.009, and right caudate, F(5, 83) = 5.481, p = 0.006,for the PTSD group to have higher RD values than the VC group, p = 0.007 and p = 0.004 respectively. The PTSD group also had a trend to have higher RD values than the TBI group, p = 0.094, in the right caudate as well.

#### AD

There were group differences in the bilateral caudate, F(5, 83) = 4.557, p = 0.013, for the PTSD group to have higher AD values than VC, p = 0.010 (Fig 1D), and a trend for the TBI group, p = 0.097. In addition, there were differences between the groups in the left caudate, F(5, 83) = 3.823, p = 0.026, and right caudate, F(5, 83) = 4.600, p = 0.013, for the PTSD group to have higher AD values than VC, p = 0.022 and p = 0.010 respectively. There were also differences between the groups in the right hippocampus, F(5, 83) = 3.581, p = 0.032, for the PTSD group to have higher AD values than the TBI group, p = 0.041.

There were no other group differences in the hippocampus for any other DTI metric (Table 3). Furthermore, the pattern of results remained consistent when Veterans who were not deployed were excluded from the analysis. In addition, normality of the DTI data was assessed and while the data was not normally distributed, the results of the log transformed data were consistent with the original analysis.

**Table 3:** Group Differences in DTI Metrics.

DTI Metrics	Veterans with PTSD (N = 25)	Veterans with TBI (N = 33)	Veterans without PTSD or TBI (N = 31)
FA			
Bilateral Hippocampus	0.20 ± 0.07	0.19 ± 0.05	0.20 ± 0.04
Left Hippocampus	0.20 ± 0.07	0.19 ± 0.05	0.20 ± 0.03
Right Hippocampus	0.20 ± 0.06	0.19 ± 0.05	0.19 ± 0.04
Bilateral Caudate	0.22 ± 0.02*	0.23 ± 0.04	0.25 ± 0.03
Left Caudate	0.23 ± 0.03	0.24 ± 0.04	0.26 ± 0.03
Right Caudate	0.22 ± 0.02	0.23 ± 0.04	0.24 ± 0.03
MD			
Bilateral Hippocampus	0.0011 ± 0.0001	0.0011 ± 0.0001	0.0011 ± 0.0001
Left Hippocampus	0.0011 ± 0.0001	0.0011 ± 0.0001	0.0011 ± 0.0001
Right Hippocampus	0.0011 ± 0.0001	0.0011 ± 0.0001	0.0011 ± 0.0001
Bilateral Caudate	0.0012 ± 0.0003*	0.0011 ± 0.0002	0.0010 ± 0.0002
Left Caudate	0.0012 ± 0.0003*	0.0011 ± 0.0002	0.0010 ± 0.0002
Right Caudate	0.0012 ± 0.0003*	0.0011 ± 0.0002	0.0010 ± 0.0002
RD			
Bilateral Hippocampus	0.0010 ± 0.0001	0.0010 ± 0.0001	0.0010 ± 0.0001
Left Hippocampus	0.0010 ± 0.0001	0.0010 ± 0.0001	0.0010 ± 0.0001
Right Hippocampus	0.0010 ± 0.0002	0.0010 ± 0.0001	0.0010 ± 0.0001
Bilateral Caudate	0.0011 ± 0.0003*	0.0010 ± 0.0002	0.0009 ± 0.0002
Left Caudate	0.0010 ± 0.0003*	0.0009 ± 0.0002	0.0009 ± 0.0002
Right Caudate	0.0011 ± 0.0003*	0.0010 ± 0.0002	0.0009 ± 0.0002
AD			
Bilateral Hippocampus	0.0013 ± 0.0001	0.0013 ± 0.0001	0.0013 ± 0.0001
Left Hippocampus	0.0013 ± 0.0001	0.0013 ± 0.0001	0.0013 ± 0.0002
Right Hippocampus	0.0014 ± 0.0001§	0.0013 ± 0.0001	0.0013 ± 0.0001
Bilateral Caudate	0.0014 ± 0.0003*	0.00013 ± 0.002	0.0013 ± 0.0002
Left Caudate	0.0014 ± 0.0003*	0.0013 ± 0.0002	0.0012 ± 0.0002
Right Caudate	0.0014 ± 0.0003*	0.0013 ± 0.0002	0.0013 ± 0.0002

Group differences in DTI metrics of Veterans with PTSD, with TBI, and without TBI or PTSD (VC).

* p < 0.05, PTSD > VC.

§ p < 0.05, PTSD > TBI.

### Volumetric group differences

There were no statistical differences between the groups in volume of the caudate and hippocampus.

### Correlation of age with MD and volume

**Fig 2:**
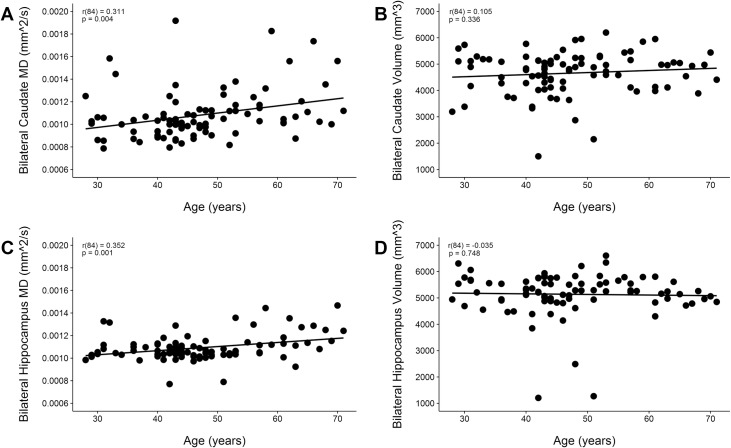
Associations between Age, MD, and Volume. Correlations between (A) Age and MD of the bilateral caudate, r(84) = 0.311, p = 0.004, (B) Age and volume of the bilateral caudate, r(84) = 0.105, p = 0.336, (C) Age and MD of the bilateral hippocampus, r(84) = 0.352, p = 0.001, and (D) Age and volume of the bilateral hippocampus, r(84) = -0.035, p = 0.748 collapsed across groups and controlling for education, handedness, and deployment.

There was a positive correlation with age and MD in the bilateral caudate and left and right caudate, *p’s* < 0.018. There was also a correlation with age and MD in the bilateral hippocampus and left and right hippocampus, *p’s* < 0.003. However, there was no correlation with age and volume in the caudate or hippocampus (Fig 2).

### Correlation of the cognitive assessment with MD and volume

There was a negative correlation with the declarative memory measure of list recall raw score and MD in the right caudate, p = 0.040. There were also negative correlations with the declarative memory measure of story recall raw scores and MD in the bilateral hippocampus (S1 Fig) and left and right hippocampus, *p’s* < 0.034, as well as the right caudate, p = 0.049. In addition, we found a positive correlation between the declarative memory measure of list learning raw scores and the volume of the left hippocampus, p = 0.011. There were no other correlations with the cognitive assessments and volume.

### MANOVA analysis

For all DTI metrics, there were no statistical differences between the ROIs for the left and right side, *p’s* > 0.05.

## Discussion

This study sought to determine whether the structural integrity among the hippocampus and caudate differed between Veterans with PTSD, TBI, and neither PTSD nor TBI. Our results demonstrated no macrostructural differences between the groups in these neuroanatomical structures. By contrast, we did observe significant alterations in microstructural DTI indices in the caudate and hippocampus. We found that the PTSD group showed worse integrity across all DTI metrics than the VC group. This finding is consistent with accumulating evidence that DTI is a useful tool to detect subtle microscopic brain tissue alterations before neuronal degradation and atrophy are detectable on a macroscopic level [37, 38].

These results provide support for subcortical microstructural abnormalities for PTSD. One possibility why we did not find results for TBI is that most of our sample consisted of individuals with mild TBI, which does limit the generalizability to moderate and severe TBI. While the majority of TBI reported by individuals fall within the mild range [76], this could be why we did not find any significant results. Future studies including moderate and severe TBI might find significant differences in microstructure when compared with PTSD and controls.

The PTSD group also performed worse than the VC and TBI groups on several measures of episodic memory, a hippocampal-mediated cognitive function, which is consistent with prior research [77, 78]. In addition, we found a correlation between the episodic memory measure of list learning with the left volume of the hippocampus across all subjects, suggesting evidence for this impairment to be reflected in the volumetric structure of the hippocampus, which is similarly supported by other studies [79, 80]. There was also a relationship between cognitive impairment in several episodic memory measures and worse gray matter subcortical integrity in both the caudate and hippocampus, also providing support for microstructural abnormalities in these structures to be related to cognitive impairment.

The result of increased MD, RD, and AD and decreased FA in the caudate in absence of significant volume atrophy suggest subtle abnormalities evident only at a microstructural level. Increased MD may be a marker of a reduced number of cells and increased interstitial space [33], while increased RD and AD are considered to be indices of myelin and axonal injuries. The significant correlation with MD and age and lack thereof with volume and age also provide support for the sensitivity of DTI to capture microscopic brain alterations. We suggest that our findings of increased caudate MD may reflect cell loss and/or impaired structural integrity in PTSD but not TBI.

There are several theories that could support the role of the caudate in the pathophysiology of PTSD in the habit-like behavioral features of the disorder. Goodman and colleagues [81] propose a cognitive neuroscience framework for the modulation of multiple memory systems in PTSD. Their framework postulates that certain symptoms of PTSD, such as flashbacks and reactivity to cues, could be due to the interactions between the different memory systems favoring striatal-dependent habit learning mechanisms. S-R learning theory also supports the role of the caudate in PTSD. S-R learning is dependent on the striatum and is a process by which a particular behavior is learned due to the association between a stimulus and response (for a review see [82]). One example of S-R learning in PTSD is the association between cues (e.g., loud noises, smells, anxiety) during the trauma and the traumatic event. Consequently, in part due to this learned association, patients with PTSD still respond to the cues in the absence of a traumatic event. Therefore, in the context of multiple memory systems and S-R learning theory, our results provide further support for the striatum to underlie some behavioral symptoms of PTSD, although other neurobiological models of PTSD also exist [83, 84].

To our knowledge, this is the first study to focus selectively on macrostructural and microstructural properties of the hippocampus and striatum in examining PTSD and TBI-related neuropathology. Nevertheless, there are some limitations. The role of PTSD symptom severity and its effect on microstructural integrity could not be determined in this study. Another limitation is that there was no cognitive assessment test that was dependent on the striatal-dependent habit memory system; it would be interesting to identify the functional role of striatal abnormalities in PTSD with respect to cognitive assessment performance in the future. In addition, this study examined structure and not function, and thus cannot make any claims about functional impairment. We also did not find any significant group differences for the TBI group; this could be due to the extreme heterogeneity of the location of injury, type of impact, TBI severity, time since injury, and age at injury. In future studies, it would be interesting to group patients with TBI into similar cohorts to see if there might be any group differences. Finally, as we did not have access to any longitudinal data, it would be important to determine if abnormalities in the caudate are present before or after the stress experience.

In summary, our DTI results and volumetric results from other studies indicate that it is important to investigate medial temporal lobe and striatal structures in PTSD, which have been understood as key targets of pathophysiological processes in this disorder. Because volume reduction in the hippocampus has been found in some studies but not all, and that microscopic brain tissue alterations are often detectable before neuronal degradation and atrophy on a macroscopic level, it seems that altered subcortical microstructure, rather than macrostructure, may be a more consistent hallmark of subcortical pathology in PTSD. Our findings support this notion. Ultimately, our findings suggest that MD is a more sensitive marker of brain tissue deficits than brain volume, consistent with other reports [37, 48]. Thus, DTI appears to be a valuable tool to investigate neuropathology in PTSD, greatly enhancing the ability to detect subtle brain changes in this complex disorder.

## Supporting Information

S1 Fig Associations between Cognitive Assessment and MD. Negative correlation between the declarative memory measure of story recall raw scores and MD in the bilateral hippocampus, r(57) = 0.305, p = 0.019 collapsed across groups and controlling for education, handedness, and deployment.
(TIF)

S1 Data This file contains the raw data for all the analyses in the manuscript. (XLSX)
