# Supplementary figures and images for: Altered Microstructural Caudate Integrity in Posttraumatic Stress Disorder but Not Traumatic Brain Injury
Source: PLoS One. 2017 Jan 23;12(1):e0170564. doi: 10.1371/journal.pone.0170564 (PMC5256941; doi:10.1371/journal.pone.0170564)

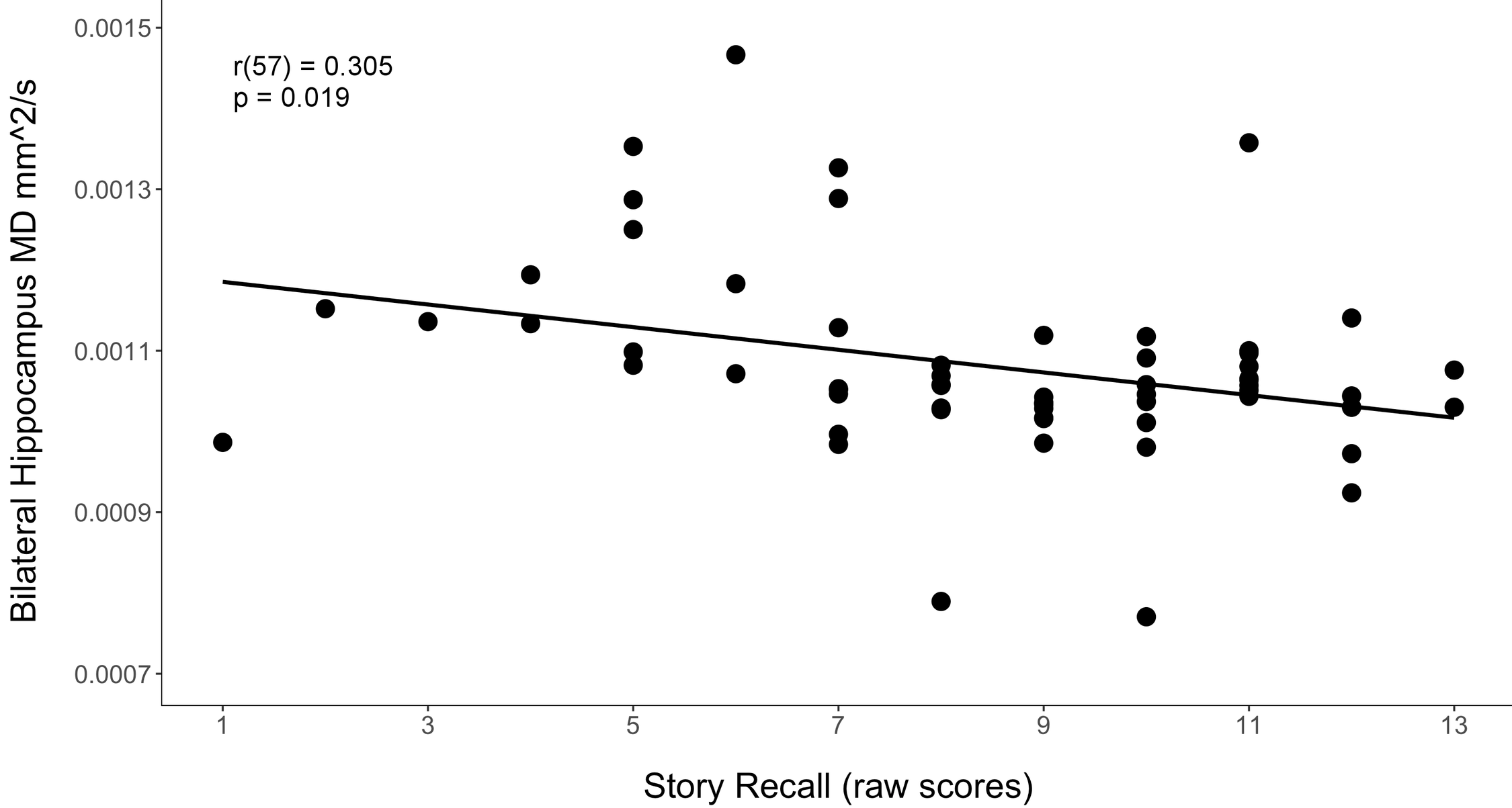

Supplement: S1 Fig — Negative correlation between the declarative memory measure of story recall raw scores and MD in the bilateral hippocampus, r(57) = 0.305, p = 0.019 collapsed across groups and controlling for education, handedness, and deployment. (TIF) [file pone.0170564.s001.tif]
